# Effects of 17‐beta estradiol on inflammation and mitochondrial function in Alzheimer’s disease

**DOI:** 10.1002/alz.091551

**Published:** 2025-01-09

**Authors:** Pranav Mishra, Ehsan Esfahani, Paul Fernyhough, Benedict C. Albensi

**Affiliations:** ^1^ Division of Neurodegenerative Disorders, St. Boniface Hospital Albrechtsen Research Centre, Winnipeg, MB Canada; ^2^ Department of Pharmacology & Therapeutics, College of Medicine, University of Manitoba, Winnipeg, MB Canada; ^3^ Department of Pharmaceutical Sciences, College of Pharmacy, Nova Southeastern University, Fort Lauderdale, FL USA

## Abstract

**Background:**

Alzheimer’s disease (AD) is a neurodegenerative disorder primarily associated with aging, but manifests as a complex interplay of multiple factors. Decline in sex‐hormones, particularly 17‐beta estradiol, is linked to the aging process. The risk for onset of AD significantly increases with aging and loss of estradiol. This is concurrent with inflammation and metabolic defects, leading to cell death. Amyloid beta (Aβ), a hallmark of AD exacerbates these processes by activating pro‐inflammatory NF‐κB, leading to sustained chronic inflammation and mitochondrial dysfunction, contributing to neurotoxicity. Estradiol imparts neuroprotection by mechanisms including the regulation of mitochondrial proteins, reduction in oxidative stress, and by decreasing inflammation. So far, our preliminary studies have demonstrated the potential of estradiol in ameliorating AD, where primary cortical neuros treated with estradiol exhibited improved mitochondrial function. For our future work, we will induce AD‐like pathology in our cell cultures by exposing them to Aβ. The objective of this study is to investigate the neuroprotective effects of estradiol against Aβ‐mediated neuroinflammation and mitochondrial dysfunction.

**Method:**

Primary cortical neurons from C57BL/6 mouse embryos were isolated and cultured under defined conditions. Glial inhibitor AraC (2uM) was used obtain pure neuronal cultures followed by estradiol treatment (10nM). Expression levels of phosphorylated AMPK (pAMPK), PGC‐1α and NF‐κB inhibitor IKbα was measured via western blotting. Culture purity was determined using immunostaining. Oxygen Consumption Rate (OCR), an established measure of mitochondrial function was evaluated in live cells using SeahorseXF24 bioanalyzer.

**Result:**

Immunocytochemical analysis confirmed that our primary neuronal cultures were >90% pure. Estradiol treatment increased both; the levels of cellular energy sensor pAMPK and master regulator of mitochondrial biogenesis PGC‐1α. Additionally, estradiol elevated ATP production and spare respiratory capacity in these cells. We also observed a decrease in pro‐inflammatory NF‐κB activation as estradiol decreased the phosphorylation and degradation of NF‐κB inhibitory protein IKbα.

**Conclusion:**

Estradiol, by elevating the levels of pAMPK, PGC‐1α, ATP production and spare respiratory capacity, and by decreasing pro‐inflammatory NF‐κB activation, significantly underlines its importance in modulating mitochondrial function and inflammation in neurons. The anticipated outcomes hold promise for advancing our understanding of therapeutic strategies in alleviating neurodegenerative diseases including AD.

